# Inhibitory influence of cationic Gemini surfactant on the dissolution rate of N80 carbon steel in 15% HCl solution

**DOI:** 10.1038/s41598-021-90031-x

**Published:** 2021-05-18

**Authors:** M. A. Deyab, Q. Mohsen

**Affiliations:** 1 Egyptian Petroleum Research Institute (EPRI), Nasr City, Cairo Egypt; 2 Department of Chemistry, College of Sciences, Taif University, Taif, Saudi Arabia

**Keywords:** Chemistry, Corrosion

## Abstract

Strong acids are commonly used in petroleum wells to remove scale layers from the surface of N80 C-steel pipe. The corrosive effects of these acids, on the other hand, pose a significant risk to C-steel pipes. For the first time, we discovered the anti-corrosion properties of cationic Gemini surfactant, 1,2-bis(dodecyldimethylammonio) ethane dibromide (DMAEB), for N80 C-steel pipe in acid washing solution (15.0% HCl). The DMAEB, in particular, can reduce the corrosion rate of N80 C-steel by approximately 97%. DMAEB molecules work as a mixed-type corrosion inhibitor, according to electrochemical results. The DMAEB demonstrated a high inhibition effect at high temperatures, as well as high activation energy against the corrosion process. DMAEB's significant performance is primarily due to physical adsorption on the N80 C-steel surface, as confirmed by adsorption isotherms, SEM, EDX, FT-IR, and theoretical studies. Our findings shed new light on the use of Gemini surfactants as corrosion inhibitors in petroleum wells.

## Introduction

Today, the use of strong acids is an important source of promoting petroleum well productivity^1^. Hydrochloric acid (HCl) is injected into the N80 C-steel pipe to remove the scale layers from the pipe surface^2^. During acid washing, the strong acid causes severe corrosion in the steel pipe wall, reducing the pipe's strength and resulting in material destruction^3, 4^. During the cleaning process, corrosion inhibitors are mixed with the acids as a first line of defence^5–8^. The most important source of corrosion protection for steel pipes is a group of various corrosion inhibitors such as organic compounds, inorganic compounds, and heterocyclic compounds^9–14^. Indeed, the toxic effects of these compounds compelled many researchers to use nontoxic alternatives to control corrosion in the petroleum industry.

Surfactants have recently been used to replace traditional corrosion inhibitors, providing minimal risk and mitigating environmental impacts^15–17^. In comparison to other reported corrosion inhibitors such as organic and inorganic inhibitors, the use of Gemini surfactants (GS) is the most practical additive due to many advantages such as low toxicity, no irritating odor, good thermal stability, and high efficacy.

Presently, GS is capable of corrosion inhibition in different media with very high efficacy^18, 19^. GS is composed of two head groups (hydrophilic) and two tails (hydrophobic) tied with the spacer^20^. Therefore, the likely role of GS in the control of corrosion will be more effective than the conventional surfactants .Moreover; the cationic GS would provide increased anti-corrosion properties due to its antibacterial effects against the bacteria in the petroleum field.

The novelty in this work is the exploring for the first time the anti-corrosion properties of cationic Gemini surfactant, 1,2-bis(dodecyl dimethylammonio) ethane dibromide (DMAEB), for N80 C-steel pipe in the acid washing solution (15% HCl). Although many works have been conducted in the field, there is still a lack in the theoretical and mechanistic approaches. In this work, we used both experimental (chemical, electrochemical and surface inspections) and theoretical approaches to explain the mechanism of inhibition efficiency of DMAEB.

## Materials and methods

### Materials

**Figure 1:**
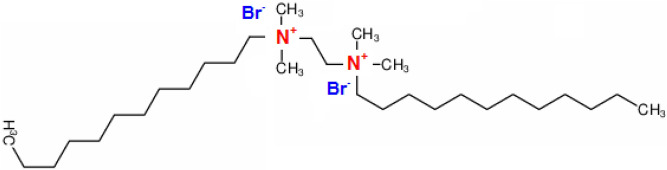
Molecular structure of 1,2-bis(dodecyldimethylammonio) ethane dibromide (DMAEB).

An Egyptian steel company supplied N80 C-steel pipe (composition: ≈ 0.33% C, 0.24% Si, 1.45% Mn, 0.05% Nb, 0.05% V, the balance Fe). Sigma-Aldrich provided the 1,2-bis(dodecyldimethylammonio) ethane dibromide (DMAEB) (purity 98%) and HCl (purity 37%). Figure 1 depicts the molecular structure of DMAEB.

### Methods

The surfaces of the N80 C-steel specimens were prepared before each experiment according to ASTM G1-03^21, 22^. ASTM G31—72(2004) standard method was used to conduct the mass loss experiments and evaluating corrosion rate^23, 24^.

The polarization experiments were recorded using a three-electrode cell (working electrode = N80 C-steel, reference electrode = SCE, counter electrode = Pt) and potentiostat instrument (EG/G Model 273A). The polarization experiments were conducted in the potential range ± 250 mV vs. OCP and using a scan rate of 1.0 mV s^−1^.

All measurements (potentiodynamic polarisation, gravimetry) were carried out three times under identical conditions. The averages of all data points were recorded.

The critical micelle concentration (CMC) of the DMAEB in the pure water was determined by surface tension measurements using Tensiometer (KRÜSS Scientific).

The surface morphology investigations (SEM and EDX) were conducted using ZEISS/EVO Scanning Electron Microscope fitted with EDX analyzer. FT-IR spectra were recorded via FT-IR spectrophotometer (Shimadzu: IRTracer™-100).

Quantum chemical calculations were studied using the VAMP module in Materials Studio-6.0-software from Accelrys Inc.

## Results and discussion

### anti-corrosion properties of DMAEB

The mass loss method and electrochemical technique (i.e. polarization test) have been used to investigate the anti-corrosion capabilities of DMAEB for N80 C-steel pipe in the acid washing solution (15% HCl).

Table 1 shows the corrosion rate (*C*_R_) from mass loss measurements, as well as the inhibition efficiency (*E*_w_%), for N80 C-steel pipe in 15% HCl solution with increasing concentrations of DMAEB at 303 K.

**Table 1:** Corrosion parameters obtained from mass loss method for N80 C-steel pipe in 15.0% HCl solution without (blank) and with DMAEB at 303 K.

DMAEB Conc mg/l	*C*_R_ average value ± standard deviation (mg/cm^2^/h)	*E*_w_%
Blank	2.84 ± 0.19	–
20	1.97 ± 0.13	30.6
40	1.02 ± 0.11	64.0
60	0.42 ± 0.05	85.2
80	0.16 ± 0.03	94.3
100	0.09 ± 0.01	96.8
120	0.10 ± 0.01	96.4
150	0.09 ± 0.01	96.8

The following relationships were used to calculate the *C*_R_ and *E*_w_%^25, 26^:

1 $$C_{\text{R}} = {\text{M}}/{\text{t}} \times {\text{A}},$$

2 $$E_{\text{W}} \% = \frac{{C_{{\text{R0}}} - C_{\text{R}} }}{{C_{{\text{R0}}} }} \times 100$$

M is the mass loss in N80 C-steel specimen, t is the time of immersion, A is the surface area, *C*_R0_ is the corrosion rate for blank solution and *C*_R_ is the corrosion rate for inhibited solution.

According to Table 1, the acid solution treatment with DMAEB resulted in a decrease in *C*_R_ values. The change in DMAEB concentrations had a significant impact on the *C*_R_ values. This means that increasing DMAEB concentrations causes a decrease in *C*_R_ values.

The addition of 100 mg/l of DMAEB results in the highest inhibition efficiency (*E*_w_% = 96.8) (Table 1). There was no significant change in the *E*_w_% value above 100 mg/l.

**Figure 2:**
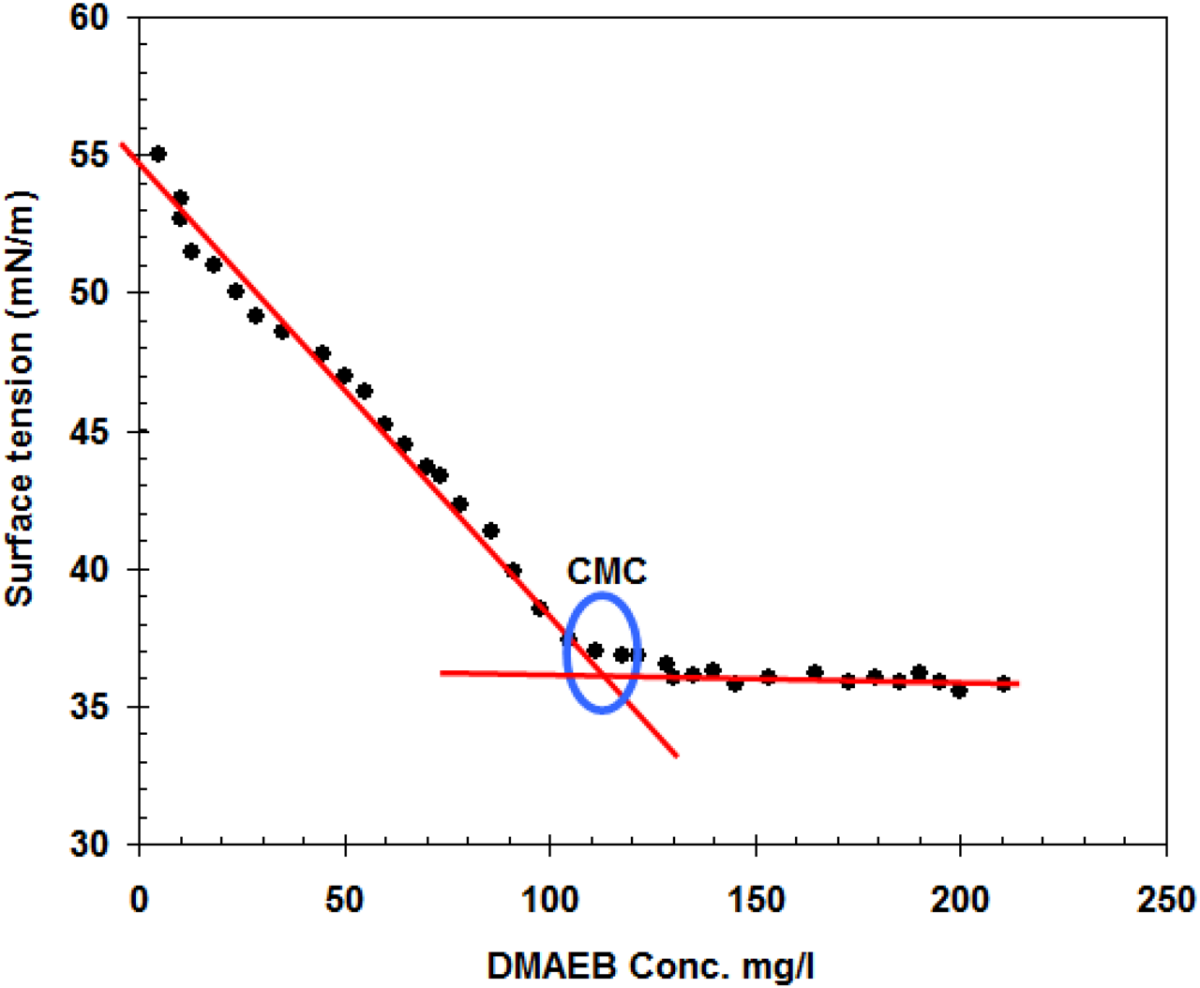
Variation of the surface tension with concentrations of DMAEB at 303 K.

Because the CMC of Gemini surfactant is important in determining a surfactant's inhibition efficiency^27^, the CMC value of DMAEB was determined by surface tension measurements, as shown in Fig. 2. According to Fig. 2, the CMC value of DMAEB is 111 mg/l. This indicates that DMAEB's maximum performance was achieved near its CMC value. At the C-steel/solution interface, a complete monolayer of DMAEB was formed at CMC value. There are no spaces available for the adsorption of additional surfactant molecules in this case. This refers to the direct relationship between the surfactant's CMC value and corrosion inhibition.

These findings show that the cationic Gemini surfactant DMAEB has a high efficacy (96.8%) in inhibiting corrosion of N80 C-steel pipe in 15.0% HCl solution at a low concentration (100 mg/l).

**Figure 3:**
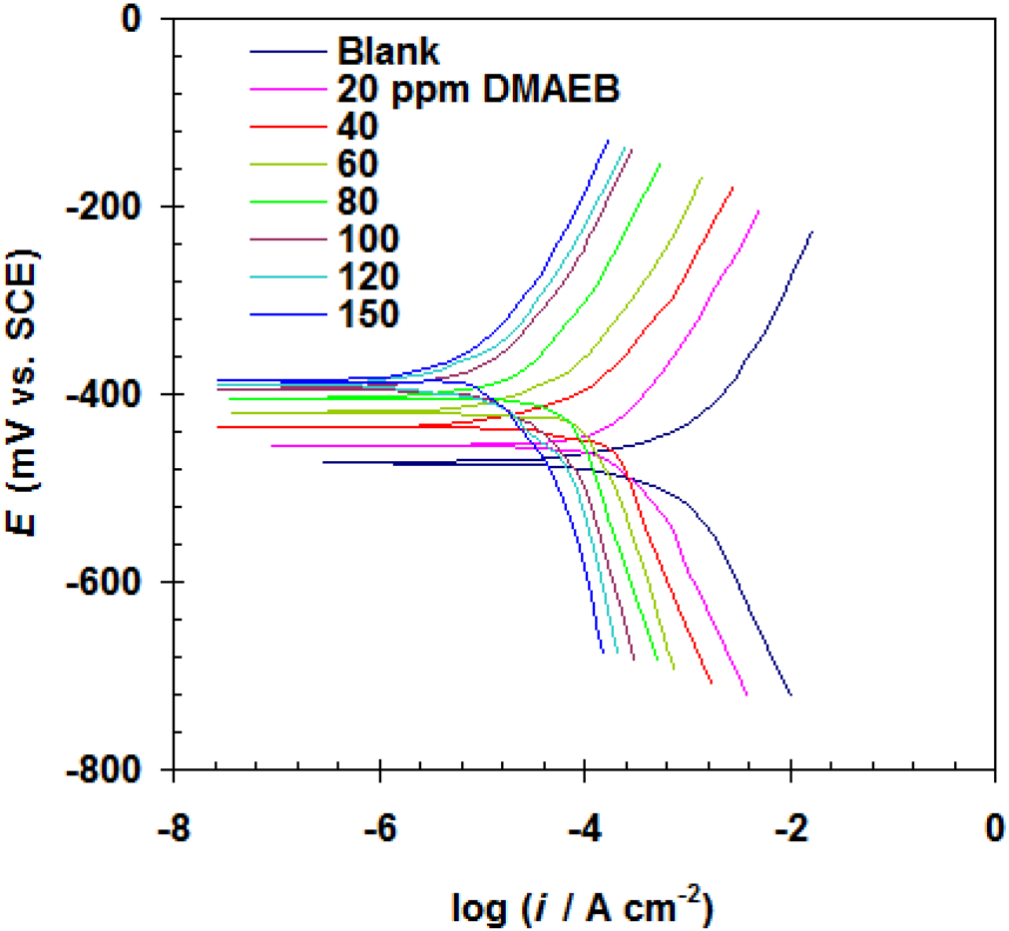
Polarization curves of N80 C-steel pipe in 15.0% HCl solution without (blank) and with DMAEB at 303 K.

The electrochemical parameters of N80 C-steel pipe were evaluated using the polarization method in both 15.0% HCl solution and blank solution treated with Gemini surfactant DMAEB. As shown in Fig. 3, the treatment of 15.0% HCl solution with DMAEB resulted in a reduction of both anodic and cathodic lines. The change in the polarization curves was found to be dependent on DMAEB concentration.

When DMAEB was added to a 15.0% HCl solution, the corrosion potential (*E*_corr._) shifted to the positive direction (Table 2). At 150 mg/l, the maximum shifting in *E*_corr._ was less than 85 mV. DMAEB was identified as the mixed-type corrosion inhibitor based on these findings^28, 29^. Furthermore, the addition of DMAEB resulted in a significant decrease in corrosion current density (*i*_corr_) (Table 2). While *i*_corr_ decreases, the changes in both Tafel line slopes (*b*_a_ for anode and *b*_c_ for cathode) remain nearly constant (Table 2). This discovery supports DMAEB's inhibitory mechanism, which involves blocking the anodic and cathodic sites by surfactant molecules^30, 31^.

**Table 2:** Electrochemical parameters obtained from polarization curves of N80 C-steel pipe in 15.0% HCl solution without (blank) and with DMAEB at 303 K.

DMAEB Conc	*E*_corr_	*b*_a_	*b*_c_	*i*_corr_	*E*_p_%
mg/l	mV (SCE)	(mV/dec)	(mV/dec)	μA/cm^2^	
Blank	− 474	75	123	623	–
20	− 455	82	120	401	35.6
40	− 435	87	112	182	70.7
60	− 419	79	133	63	89.8
80	− 404	89	143	20	96.8
100	− 398	85	127	17	97.2
120	− 394	92	128	16	97.4
150	− 390	96	126	15	96.5

The following relationship can be used to calculate the inhibition efficiency *E*_p_% values based on polarization measurements^32, 33^:

3 $$E_{\text{P}} \% = \frac{{i_{{\text{corr(0)}}} - i_{{\text{corr}}} }}{{i_{{\text{corr(0)}}} }} \times 100.$$

*i*_corr(0)_ is the corrosion current density for blank solution).

The increase in DMAEB concentration is directly proportional to *E*_p_% values. The highest DMAEB concentration, 100 mg/l, resulted in a 97% reduction in corrosion rate (Table 2).

**Figure 4:**
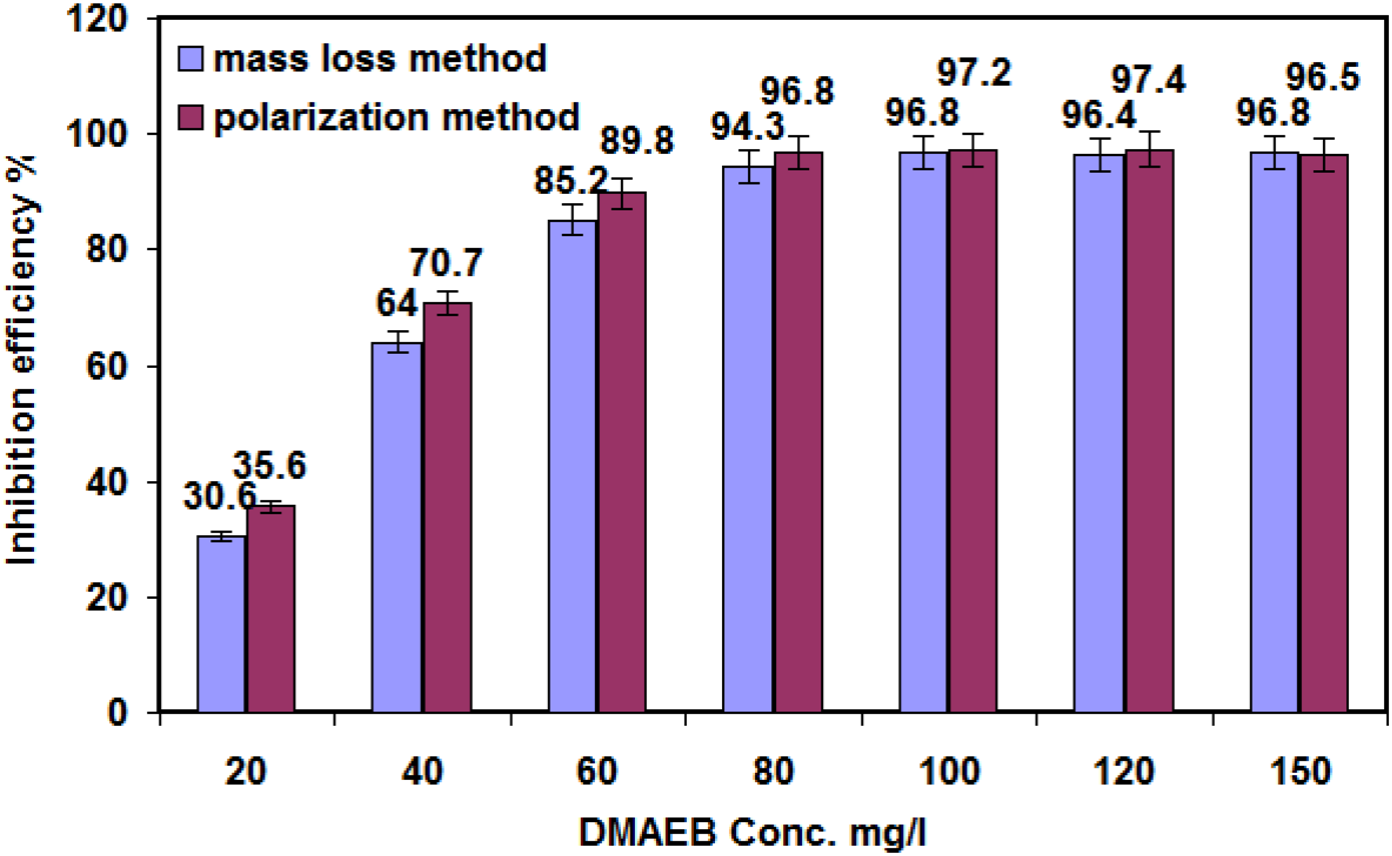
Comparison of inhibition efficiency% values obtained from mass loss and polarization methods.

Figure 4 depicts a histogram comparing the inhibition percentages of the two techniques (polarization and mass loss) for the same conditions. It appears that no significant differences in inhibition percentage values are observed regardless of the techniques used. The inhibition percentages obtained by mass loss measurements were found to be slightly lower than those obtained by polarization measurements. This is because the surface of N80 C-steel is exposed to the acidic solution for a longer period of time when using the mass loss method^34^.

**Figure 5:**
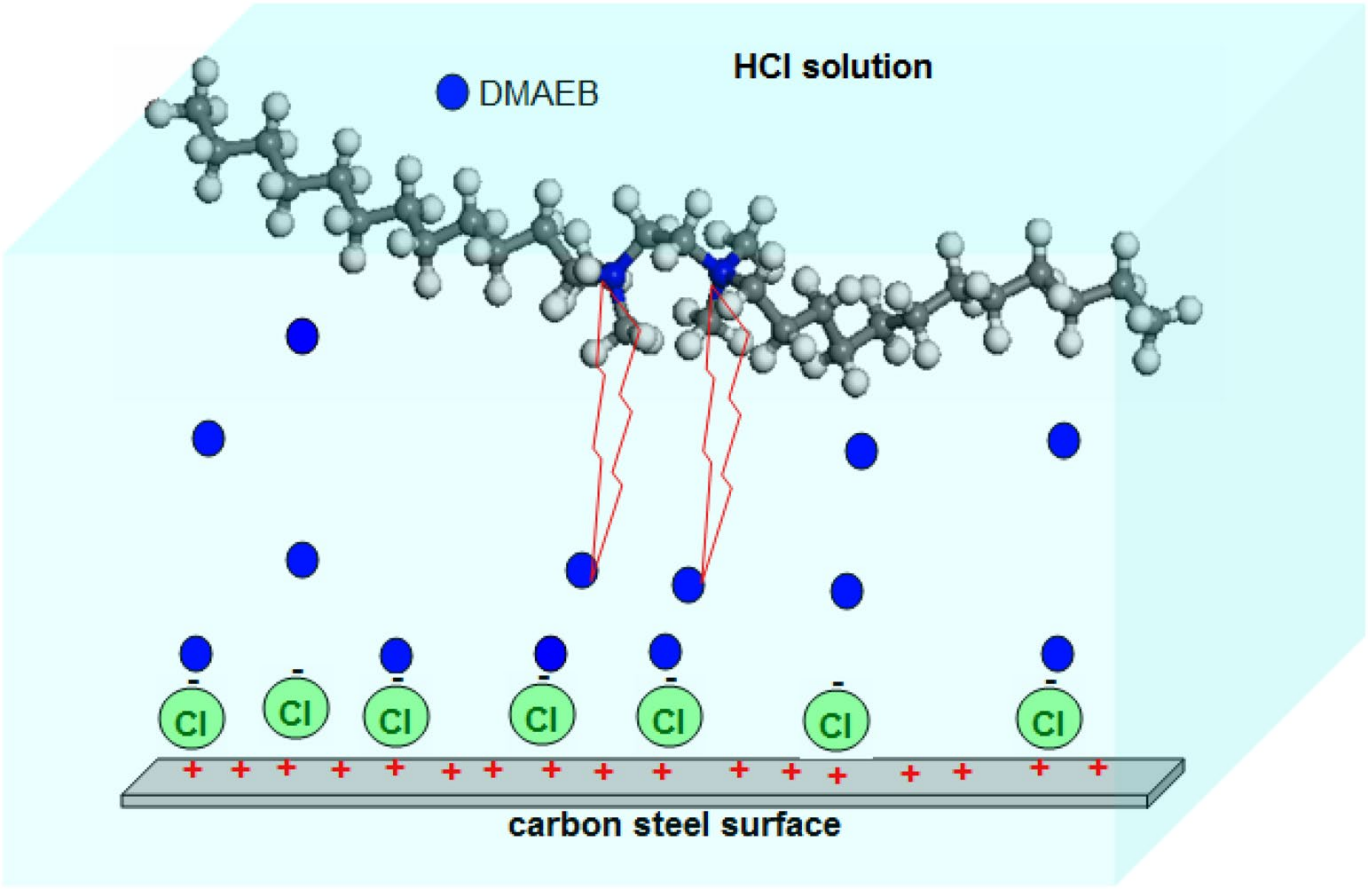
Schematic illustration of inhibition mechanism of DMAEB for N80 C-steel corrosion in 15.0% HCl solution.

The main mechanism of the Gemini surfactant's anti-corrosion properties is based on its adsorption capability on the surface of the N80 C-steel pipe^35, 36^. Because the surface of the steel in acidic solution has a positive charge, the adsorption of Cl^-^ ions from the solution on the steel surface is facilitated^37^. The steel surface was changed to a negatively charged surface in this case. This situation promotes electrostatic interactions between positively charged DMAEB molecules and the steel surface, resulting in the formation of the DMAEB surface layer. This surface layer protects the N80 C-steel pipe's surface from the corrosive acid solution, resulting in a low corrosion rate^38^. The short spacer group connects the two head groups in GS. This facilitates the hydrophobic interaction between the DMAEB's two alkyl tails, lowering the CMC value. Furthermore, the short spacer group promotes the formation of more rigid molecules. This prevents DMAEB molecules from desorbing from the metal surface, resulting in increased inhibition efficiency^39^. Additionally, the long two alkyl chains help to cover the surface of the N80 C-steel pipe^40^. The schematic illustration of the inhibition mechanism is shown in Fig. 5.

DMAEB has a higher efficiency in a more corrosive solution when compared to previous works for other corrosion inhibitors of the same family in the literature^41–45^ (see Table 3).

**Table 3:** Comparison of the inhibition efficiency of DMAEB with other inhibitors of the same family for carbon steel in HCl solution reported in the literature.

Compounds	Conc	Temperature	Solution	Efficiency	References
	M	K		%	
1,4-bis (1-Chlorobenzyl-benzimidazolyl)-butane	3.7 × 10^−4^	303	0.5 M HCl	89.7	^41^
2,2′-Bipyridyl-5,5′-dimethylene-bis(*N*,*N*′-dimethylalkylammonium bromide)	1 × 10^–4^	303	1 M HCl	83.4, 88.1, 88.6	^42^
*n* = 8, 10, and 12					
Propanediyl-1,3-bis(*N*,*N*-dihydroxyethyl-*N*-dodecylammonium bromide)	3.0 × 10^–5^	298	1 M HCl	88.5	^43^
Pyridyl gemini surfactants	≈ 1 × 10^−3^	303	1 M HCl	90.33	^44^
bis-Quaternary ammonium salt gemini surfactan	3.28 × 10^−^	298	1 M HCl	90	^45^
DMAEB	1.62 × 10^−4^	303	15% HCl	≈ 97	This work

### Temperature dependence and activation energy

The *C*_R_ values of the N80 C-steel pipe in 15.0% HCl solution containing 100 mg/l of DMAEB were recorded at temperatures ranging from 303 to 333 K to predict the stability of Gemini surfactant DMAEB at high temperatures (Table 4). The highest temperature (333 K), as shown in Table 4, reduced the *E*_w_% of DMAEB to 92.5%. This finding confirms DMAEB's high stability at high temperatures. Two factors contribute to the slight decrease in *E*_w_% of DMAEB at high temperatures. The first factor is an increase in the corrosion rate of steel as temperature rises^46^. The second factor is the high-temperature desorption of some DMAEB molecules from the steel surface (i.e. physical adsorption)^47^.

**Table 4:** Corrosion parameters obtained from mass loss method for N80 C-steel pipe in 15% HCl solution without and with 100 mg/l DMAEB at different temperatures.

Temperature (K)	Solution	*C*_R_ (mg/cm^2^/h)	*E*_w_%
303	15% HCl	2.84 ± 0.19	–
	15% HCl + 100 mg/l DMAEB	0.09 ± 0.01	96.8
313	15% HCl	2.95 ± 0.19	–
	15% HCl + 100 mg/l DMAEB	0.12 ± 0.01	95.9
323	15% HCl	3.23 ± 0.19	–
	15% HCl + 100 mg/l DMAEB	0.17 ± 0.02	94.7
333	15% HCl	4.68 ± 0.19	–
	15% HCl + 100 mg/l DMAEB	0.35 ± 0.03	92.5

The relationship between corrosion rate *C*_R_ and activation energy (*E*_a_) is expressed by the Arrhenius formula (see Eq. 4) ^48^.

4 $$C_{R} = A\exp \left( {\frac{{ - E_{a} }}{RT}} \right).$$

**Figure 6:**
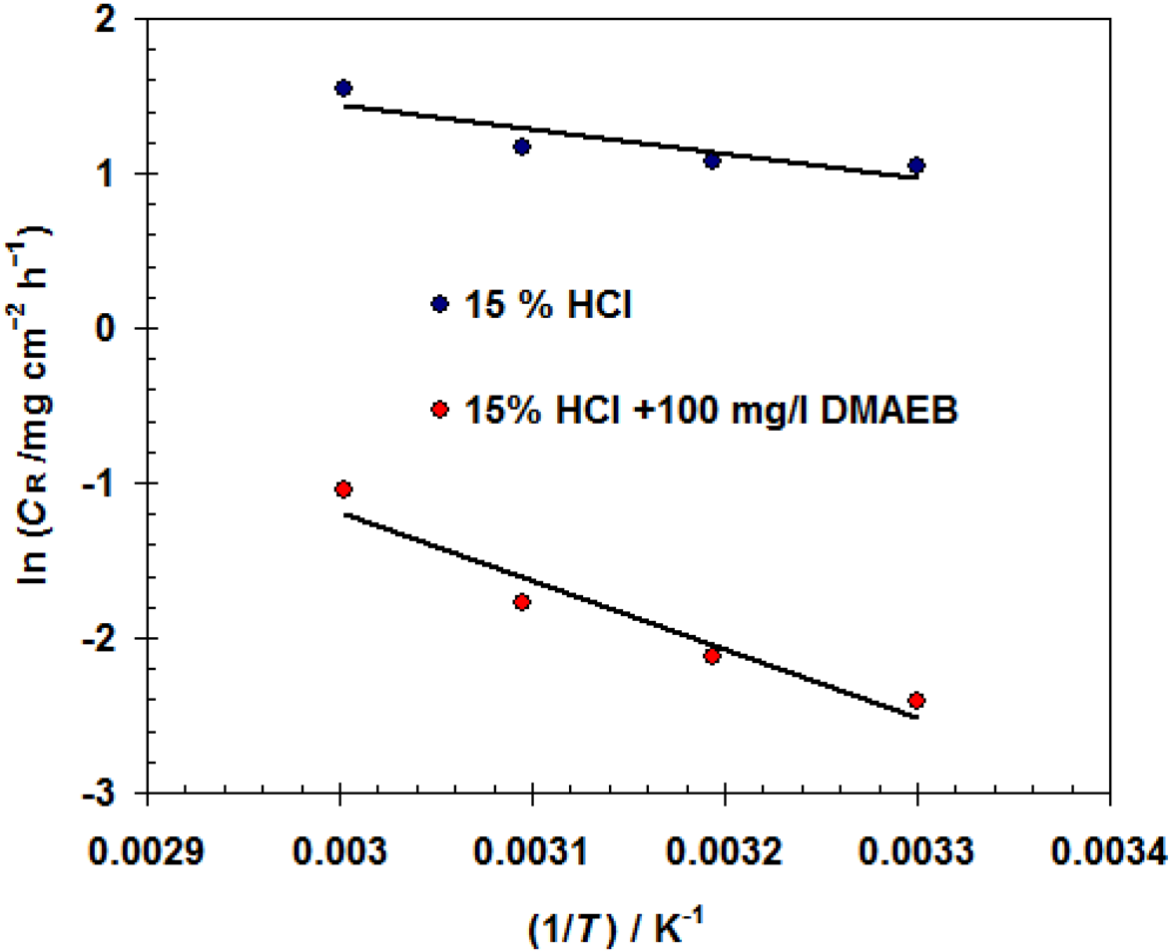
Arrhenius plot for N80 C-steel pipe in 15.0% HCl solution without and with 100 mg/l DMAEB.

The *E*_a_ was calculated using the Arrhenius plot (straight-line gradient) (see Fig. 6).

The calculated *E*_a_ for the corrosion reaction in 15.0% HCl solution containing 100 mg/l of DMAEB is 36.74 kJ/mol, which is comparable to the calculated *E*_a_ in 15.0% HCl solution (13.08 kJ/mol). This finding confirms that the presence of DMAEB in a 15.0% HCl solution raises the energy barrier for corrosion, resulting in a low corrosion rate^49–51^.

### Adsorption isotherm studies

Because the effectiveness of Gemini surfactant DMAEB is dependent on DMAEB molecules' adsorption capability on the steel surface, it is critical to investigate the adsorption isotherm in this section. The Langmuir adsorption isotherm (Eq. 5) is the best fitting isotherm that describes the adsorption process of DMAEB molecules based on mass loss measurements^52^.

5 $$\frac{{C_{{\text{inh}}} }}{\theta } = \frac{1}{{K_{{\text{ads}}} }} + C_{{\text{inh}}} .$$

*θ* is the surface coverage = *E*_w_%/100, *C*_inh_ is the DMAEB concentration, *K*_ads_ is the equilibrium constant).

**Figure 7:**
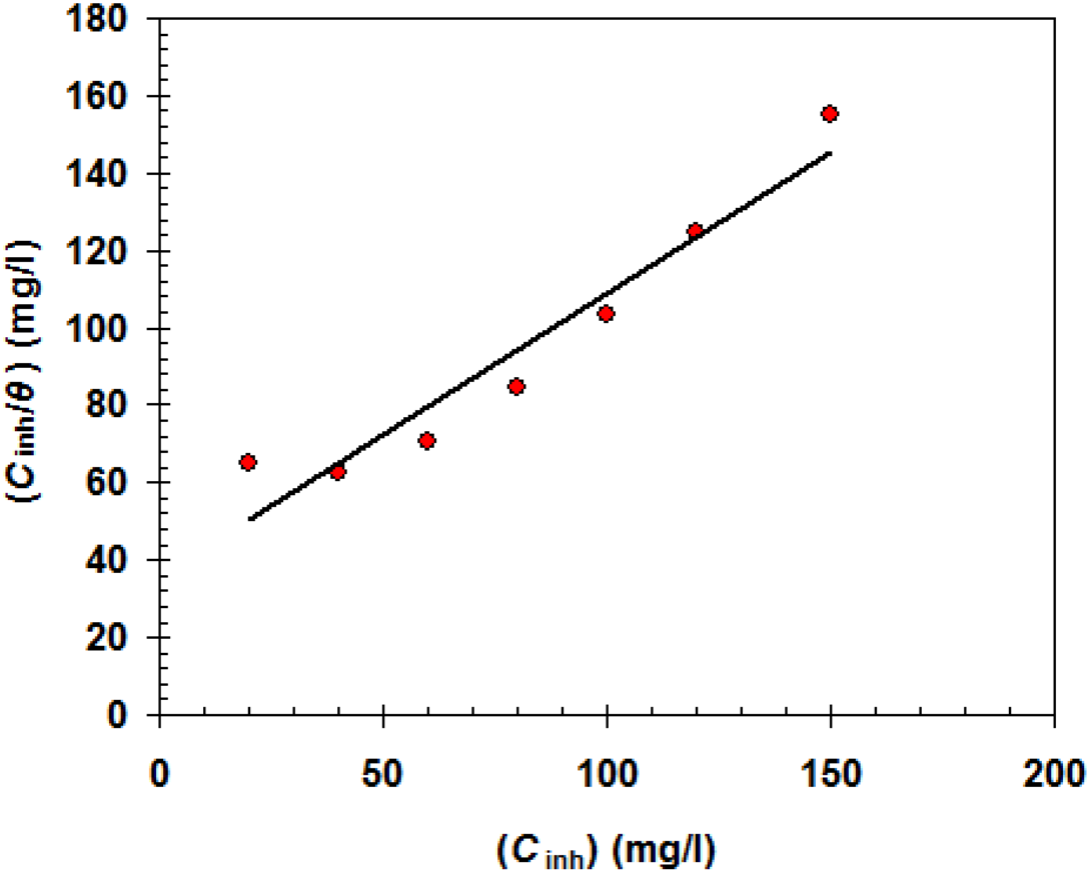
Langmuir isotherm plot for N80 C-steel pipe in 15.0% HCl solution containing 100 mg/l DMAEB at 303 K.

The linear correlation coefficient (R^2^) is very close to one in the Langmuir isotherm plot (Fig. 7), confirming the validity of this isotherm^53^. The DMAEB's *K*_ads_ was determined to be 1.72 × 10^4^ M^−1^.

Gibbs free energy change (∆G°_ads_) was calculated from the following relation^54^:

6 $$\Delta {\text{G}}^\circ_{{{\text{ads}}}} \, = - RT{\text{ln }}\left( {{55}.{5}K_{{{\text{ads}}}} } \right).$$

The ∆G°_ads_ for the DMAEB was identified to be − 34.62 kJ/mol. A negative ∆G°_ads_ value confirms the spontaneity of DMAEB adsorption on the steel surface^55^. The value of ∆G°_ads_ (i.e. − 34.62 kJ/mol) refers to DMAEB physisorption on the surface of the N80 C-steel^56^.

### Surface analysis

**Figure 8:**
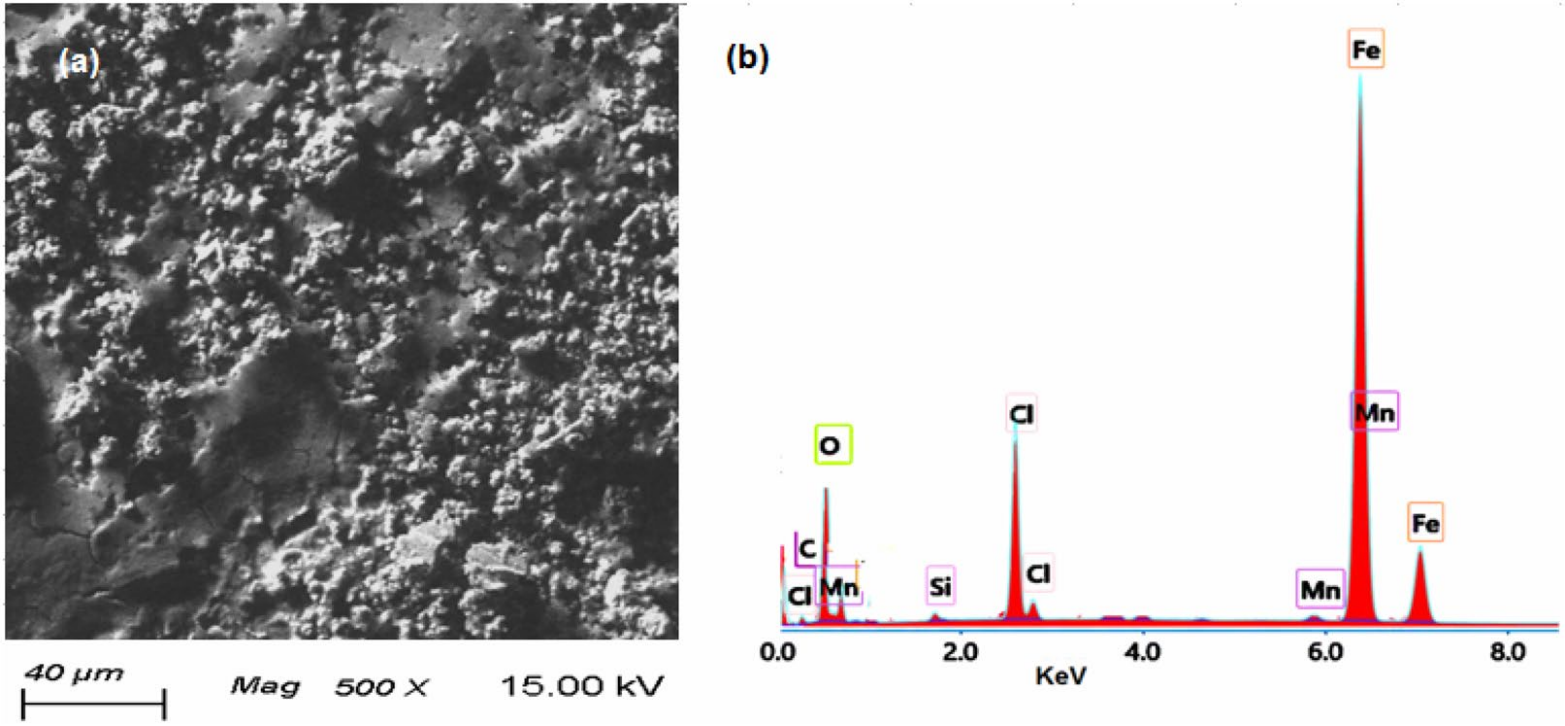
(**a**) SEM image and (**b**) EDX spectra for N80 C-steel pipe after immersion in the blank solution (15.0% HCl).

**Figure 9:**
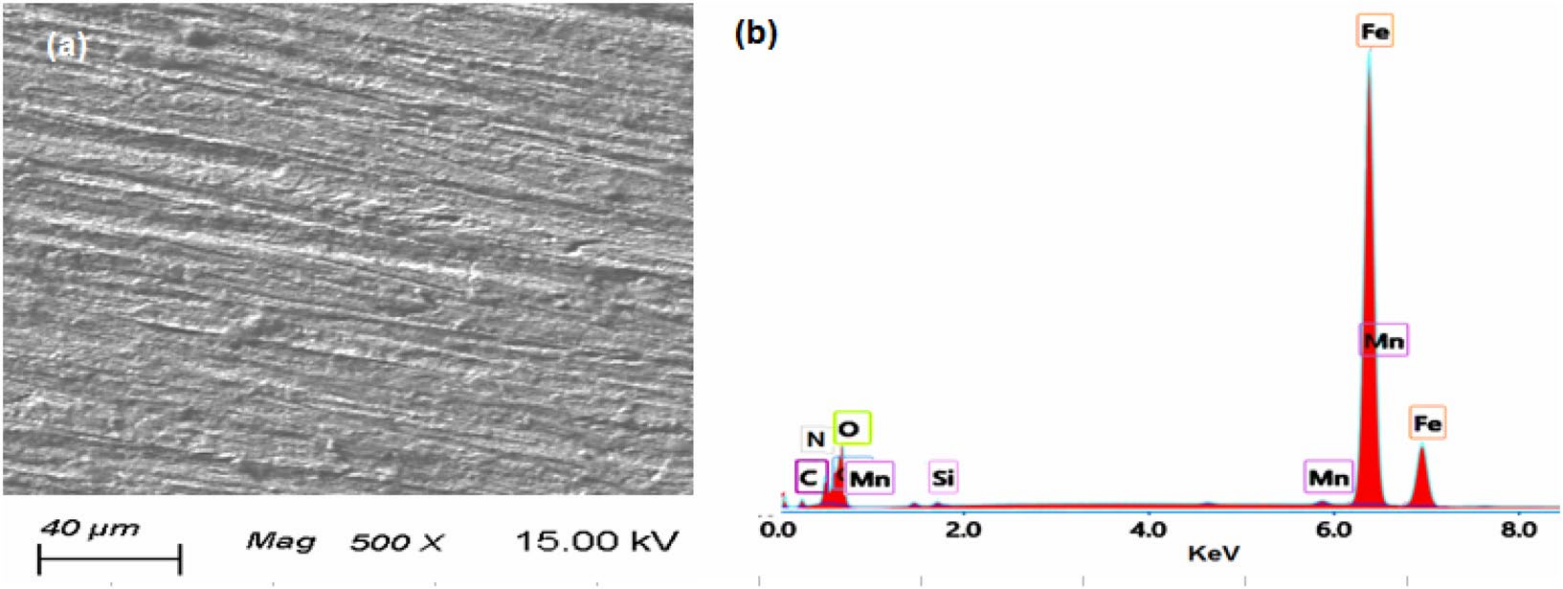
(**a**) SEM image and (**b**) EDX spectra for N80 C-steel pipe after immersion in the inhibited solution (15.0% HCl + 100 mg/l DMAEB).

The morphological analysis (SEM and EDX) of the N80 C-steel in the blank solution (15.0% HCl) and inhibited solution (15.0% + 100 mg/l DMAEB) are presented in Figs. 8 and 9. In the blank solution, the surface morphology of the N80 C-steel revealed a damaged structure and dense surface roughness (Fig. 8a). The N80 C-steel EDX spectrum in the blank solution (Fig. 8b) revealed characterized signals for N80 C-steel composition and corrosion products (i.e. O, Cl, C, Mn, Si, and Fe).

In the presence of 100 mg/l DMAEB, the N80 C-steel has a smooth surface and is free of corrosion products (Fig. 9a). The presence of characterized DMAEB signals is revealed by the EDX spectrum of N80 C-steel in inhibited solution (Fig. 9b) (i.e. N and C). Furthermore, Cl signals have vanished.

**Figure 10:**
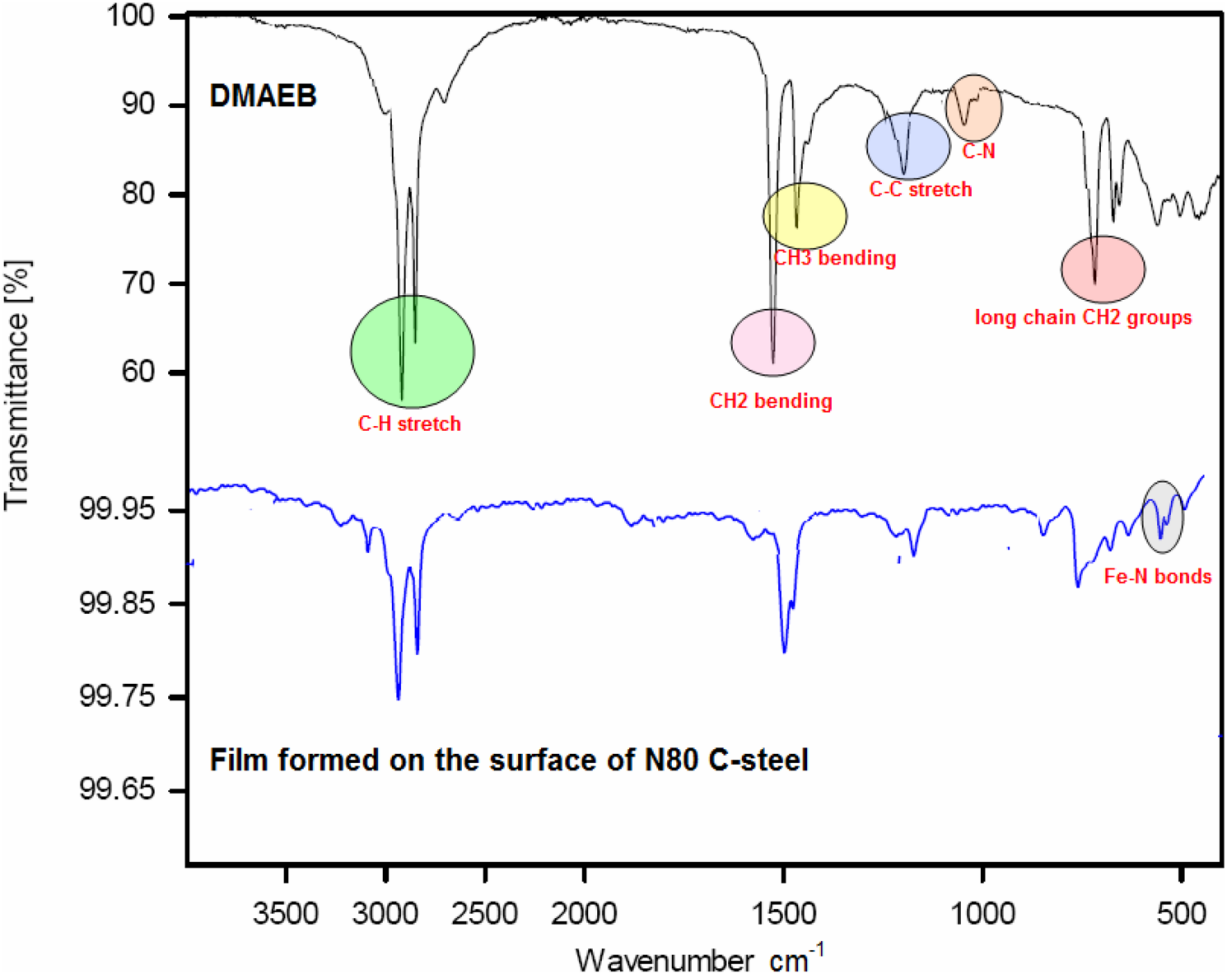
FT-IR spectra of pure DMAEB and film formed on the surface of N80 C-steel in the inhibited solution (15.0% HCl + 100 mg/l DMAEB).

The FT-IR spectra of pure DMAEB and the film formed on the surface of N80 C-steel in the inhibited solution (15.0% HCl + 100 mg/l DMAEB) were analyzed and shown in Fig. 10. The characteristic peaks for C–H stretch, CH_2_ bending, CH_3_ bending, C–C stretch, C–N, and long-chain CH_2_ groups can be seen in the FT-IR spectrum of pure DMAEB.

The FT-IR spectra of the film formed on the surface of N80 C-steel in the inhibited solution showed three changes. The first is a shift in some peaks, such as C–H stretch, CH_2_ bending, CH_3_ bending, and C–C stretch. The second change is the absence of C–N. The presence of new peaks related to Fe–N bonds at 645–450 cm^−1^ is the third change. All of these studies support the adsorption of DMAEB molecules on N80 C-steel.

### Quantum chemical calculations

**Figure 11:**
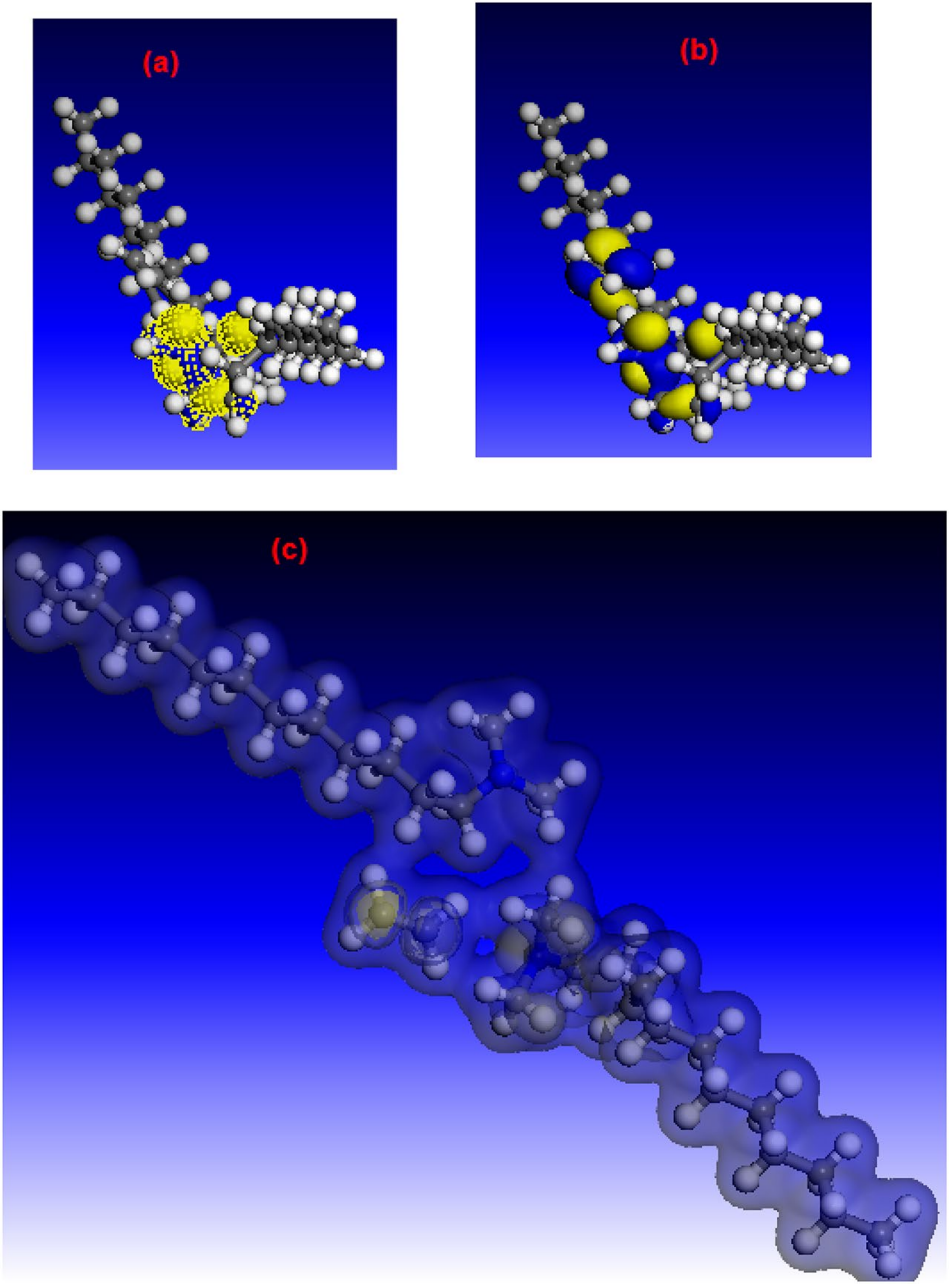
(**a**) HOMO, (**b**) LUMO and (**c**) total electron density distribution for DMAEB molecule. "Computational results obtained using software programs from Accelrys Software Inc. The ab initio calculations were performed with the DMol^3^ program, and graphical displays generated with Materials Studio".

Quantum chemical calculations were used to support the experimental results. Figure 11a,b show that HOMO (highest occupied molecular orbital) and LUMO (lowest unoccupied molecular orbital) regions are concentrated on ammonium groups. This demonstrates that the ammonium groups in DMAEB molecules are the active components in the adsorption process^31^. The high value of the HOMO energy (*E*_HOMO_ = 8.939 eV) refers to the DMAEB molecule's ability to link with the steel surface^57^. Also, the low LUMO energy (*E*_LUMO_ = 1.577 eV) refers to the DMAEB molecule's ability to gain electrons from the filled Fe d-orbital^58^. Furthermore, the low energy gap (ΔE = *E*_LUMO_ − *E*_HOMO_, 7.362 eV) refers to DMAEB molecules' high inhibition performance^59^. It was discovered that the electron density was distributed throughout the entire DMAEB molecule (see Fig. 11c). This means that DMAEB molecules are adsorbing on the Fe surface in flat-lying orientations^60^.

DMAEB's high dipole moment (i.e. μ = 6.94 Debye) indicates a strong electrostatic interaction between DMAEB molecules and the C-steel surface^61^.

The electronegativity (*χ*) and global hardness (η) parameters for DMAEB are calculated from the following relations:

7 $$\chi = \, 0.{5} \times \left( {{\text{I}} + {\text{A}}} \right),$$

8 $$\eta = \, 0.{5} \times \left( {{\text{I}} - {\text{A}}} \right),$$

where I is the ionization potential = − *E*_HOMO_ and A is the electron affinity = − *E*_LUMO_.

The calculated values of *χ* and η are 5.258 eV and 3.681 eV, respectively.

The high χ value for DMAEB molecules indicates a high ability to attract electrons and, as a result, a high adsorption efficiency^62^. Furthermore, the low η value for DMAEB molecules indicates a strong interaction between the metal surface and inhibitor molecules^63^.

## Conclusions

In summary, we investigated the anti-corrosion properties of cationic Gemini surfactant, 1,2-bis(dodecyldimethylammonio) ethane dibromide (DMAEB), for N80 C-steel pipe in the acid washing solution (15% HCl).

It is worth noting that the Gemini surfactant DMAEB has a high efficacy (96.8%) in inhibiting corrosion of N80 C-steel pipe in 15.0% HCl solution at a low concentration (100 mg/l). DMAEB's anti-corrosion properties were investigated using mass loss, polarization, SEM, EDX, and FT-IR tools. DMAEB acts as a mixed-type corrosion inhibitor, as evidenced by the polarization curves. The high temperature (333 K) slightly reduced DMAEB efficacy to 92.5%, confirming DMAEB's high stability at high temperatures. Furthermore, the presence of DMAEB in a 15.0% HCl solution raises the energy barrier for corrosion, resulting in a low corrosion rate. The Langmuir adsorption isotherm accurately described the adsorption of DMAEB molecules. The ∆G°_ads_ was − 34.62 kJ/mol, indicating physisorption behavior. SEM, EDX, and FT-IR analysis confirmed the adsorption of DMAEB molecules on the surface of N80 C-steel. To back up the experimental findings, quantum chemical calculations were used.
